# Charting a Course towards Resilience Following Adverse Childhood Experiences: Addressing Intergenerational Trauma via Strengths-Based Intervention

**DOI:** 10.3390/children8100844

**Published:** 2021-09-24

**Authors:** Kathryn H. Howell, Laura E. Miller-Graff, Cecilia Martinez-Torteya, Taylor R. Napier, Jessica R. Carney

**Affiliations:** 1 Department of Psychology, University of Memphis, Memphis, TN 38152, USA; trschwr1@memphis.edu; 2 Department of Psychology, University of Notre Dame, Notre Dame, IN 46556, USA; lmiller8@nd.edu (L.E.M.-G.); jcarney7@nd.edu (J.R.C.); 3 Kroc Institute for International Peace Studies, University of Notre Dame, Notre Dame, IN 46556, USA; 4 Department of Education, Universidad de Monterrey, San Nicolas de los Garza 66238, Mexico; cecilia.martineztorteya@udem.edu

**Keywords:** adversity, resilience, intervention science, multigenerational, family systems

## Abstract

Early research on adverse childhood experiences (ACEs) provided staggering evidence of the significant ramifications of ACEs on physical health and functioning. It brought to the forefront the importance of addressing trauma and family dysfunction to enhance public health. Over the past several decades, the study of childhood adversity has blossomed, with expanded conceptualizations and assessments of ACEs. This review brings together various biological, psychological, and sociological principles that inform our understanding of ACEs and our approach to treatment. Specifically, we document the evolution of ACEs research, focusing on the intergenerational impact of ACEs, the importance of incorporating a resilience framework when examining ACEs, and implementing interventions that address adversity across generations and at multiple levels of the social ecology. Evidence is provided to support the evolving perspective that ACEs have long-lasting effects beyond the ACE(s)-exposed individual, with significant attention to the impact of parental ACEs on child development. An intergenerational and multilevel approach to understanding and addressing ACEs offers specific areas to target in interventions and in public policy.

## 1. Introduction

Understanding the deleterious effects of exposure to adversity in childhood has been central to the study of psychology for nearly a century [1]. The earliest literature focused overwhelmingly on the consequences of experiencing single types of adversity, such as abuse or neglect by a caregiver [2]. Felitti and colleagues built upon this work and expanded the conceptualization of adversity to systematically account for the compounding effects of co-occurring adversities [3]. The landmark adverse childhood experiences (ACEs) study constituted a paradigm shift by bringing to the forefront the cumulative and interacting effects of multiple types of childhood adversities [3].

This review charts psychology’s evolution from an emphasis on the individual health effects of ACEs to frameworks that examine ACEs’ intergenerational consequences. We first discuss the history of Felitti’s original conceptualization of ACEs, which includes child maltreatment (i.e., caregiver physical, sexual, and psychological abuse, physical and emotional neglect) and household dysfunction (i.e., caregiver substance use, mental illness, incarceration, divorce, and exposure to familial intimate partner violence (IPV)). After examining this foundational work, we shift to current trends in ACEs research, with a focus on the intergenerational effects of ACEs and the cascading impact of adversity across generations. We provide a detailed overview of the consequences of maternal ACE(s) exposure, including the biological and environmental facets of intergenerational ACEs transmission. The diverse trajectories of functioning following exposure to childhood adversity are examined, including how resilience manifests at the individual, relational, and contextual levels. Finally, we discuss ameliorating the negative impact of ACEs and cultivating resilience in the context of intergenerational interventions across development. We conclude by addressing controversies in the ACEs literature and actionable steps forward.

## 2. Original ACEs Conceptualization and Associated Outcomes

The ACEs study was conducted at a Kaiser Permanente medical center and consisted of a sample of 9508 adults who received a standardized medical evaluation and completed a questionnaire about their exposure to childhood adversity [3]. Felitti and colleagues investigated the rates and long-term ramifications of child maltreatment and household dysfunction in this sample. The prevalence of ACEs in Felitti and colleagues’ study raised awareness about the pervasiveness of childhood adversity in the United States [3]. Most participants (52%) had experienced one or more types of adversity, and 6.2% reported four or more types. The study also demonstrated that experiencing one ACE was a strong predictor of experiencing at least one additional ACE. These findings established the need for investigation into the outcomes of exposure to multiple, rather than single, adversities.

In addition to raising awareness about the co-occurrence and pervasiveness of adversity, the ACEs study was also among the first to demonstrate a relationship between childhood adversities and physical health difficulties in adulthood, including heart disease, cancer, stroke, and chronic pulmonary disease [3]. The analyses demonstrated a strong dose–response relationship between the number of ACEs experienced and negative physical health outcomes, which has been replicated in numerous follow-up studies [4,5]. Additional work expanded the scope of outcomes explored, documenting increased risk for psychiatric disorders and interpersonal violence as ACEs increase in number [6].

A number of explanations were put forth to explain ACEs’ wide-ranging and enduring effects. Initial explanations focused on the contribution of ACEs to risky behaviors such as overeating, high-risk sexual activities, and substance use [3]; it was theorized that these behaviors may be repeatedly employed in an attempt to cope with enduring adversity, contributing to adult disease burden. More recent evidence shows that ACEs also directly shape the structure and function of developing neural, immune, and neuroendocrine systems, and they act as biological mechanisms of transmission, with significant consequences for physical and psychological health and disease [7]. A large body of research documents the biological correlates of direct exposure to ACEs, including alterations in prefrontal cortex volume [8], immune system functioning [9], and stress response systems (the Hypothalamic–Pituitary–Adrenal (HPA) axis and Autonomic Nervous System) [10,11]. Notably, emerging epigenetic research suggests that biological, environmental, and behavioral influences transact over time and shape pathways to health and disease among ACEs-exposed individuals [12]. Thus, multiple mechanisms are at play, with behavioral, neuroimmunological, and environmental factors interacting to inform the functioning of individuals experiencing ACEs.

## 3. Intergenerational Transmission of ACEs

In line with the “intergenerational hypothesis”, which posits that the experiences of one generation can influence the development of the next [13], the extant literature demonstrates that ACEs are not solely contained within one individual’s experience but rather have a cascading impact across generations [14,15,16,17]. Most research has focused on the effects of maternal exposure to ACEs, emphasizing pregnancy and gestation as a sensitive period during which mother and fetus are inextricably connected, marking the beginning of ACEs-dependent altered development [18]. Research has shown direct effects of maternal ACEs that begin before birth and continue throughout childhood and adolescence, including low birth weight and premature delivery [19]; enduring physical health problems [20]; poor socioemotional functioning [21]; communication, cognitive, and motor developmental delays in infants [18]; parent–child relationship difficulties in early childhood [22]; and behavioral and emotional problems throughout development [15,16,17,22]. Although the consequences of fathers’ ACEs exposure are substantially less understood than mothers’, a small body of research suggests similar effects on children’s risk for developmental delay [14], physical health challenges [20], and subsequent behavioral problems [23].

Biological parents are most often the focus of intergenerational research, but ACEs experienced by other caregivers (i.e., grandparents, aunts, uncles) also impact future generations. In one study that included other caregivers in addition to biological parents, other caregiver ACEs increased the risk for symptoms of anxiety and depression among adolescents, and those adolescents who were living with other caregivers were twice as likely to report symptoms as their peers living with at least one biological parent [24]. Notwithstanding these notable findings, research is sparse regarding the intergenerational transmission of ACEs from fathers, second parents, and other caregivers to subsequent generations. In sum, ACEs have long-lasting effects beyond the ACE(s)-exposed individual, with evidence of intergenerational impacts connected to caregiver ACEs exposure.

## 4. Biological Factors That Facilitate Intergenerational Transmission of ACEs

The intergenerational ACEs literature has primarily focused on maternal ACEs as specific mechanisms that may uniquely facilitate the transmission of adversity, such as physiological processes occurring in utero [18]. Preliminary work suggests that maternal ACEs-induced immune and endocrine alterations disrupt hormone levels in the placenta [25,26], ultimately impacting fetal development [12,27]. The effects of maternal stress response alterations during pregnancy likely extend well after gestation; one study found that maternal HPA axis functioning during pregnancy mediated the association between maternal ACEs and infant cortisol response [28]. Further, alterations in offspring brain volume and stress response systems during early childhood resemble those of individuals directly exposed to ACEs [29,30,31]. Maternal maltreatment history is associated with dampened baseline cortisol levels [30] and heightened cortisol reactivity [28,32] in infants, suggesting critical deviations in the early calibration of the HPA axis and potential risk for neurotoxic effects on the brain [33]. More maternal ACEs are also associated with the suppression of parasympathetic activity during early childhood [31,34]. This chronically high physiological arousal reflects poor emotion regulation [34,35] and, consequently, increased temperamental and behavioral problems in young children [36,37]. Moreover, direct ACEs exposure changes patterns of methylation throughout the lifespan [38] and these fluctuations may be passed on to the next generation via alterations in maternal and paternal germ cells [27].

## 5. Intergenerational Transmission of ACEs via Caregiver Mental Health and Parenting

Environmental pathways of risk transmission may also explain the intergenerational effects of ACEs. Evidence suggests that maternal childhood experiences of interpersonal trauma are strong predictors of parenting stress, maternal hostility, and insecure mother–infant attachment [18,39], which have been shown to mediate the effects of maternal ACEs on child development [22,23]. Further, mental health symptoms (i.e., depression, anxiety) in mothers with high numbers of ACEs also impact children’s development and contribute to poor social and emotional outcomes [40]. Specifically, women exposed to four or more ACEs are more likely to experience depression and anxiety, which has been linked with poor mental health outcomes in their children [24]. Children of ACEs-exposed parents are at higher risk for similar ACEs exposure [41,42], and subsequent disruptions to their physical, cognitive, and socioemotional development [43,44]. In contrast, protective factors within the mother–child relationship, such as positive parenting, may bolster adaptive outcomes for children with ACEs-exposed mothers [23], highlighting the importance of enhancing parenting capacities within interventions aimed at ending cycles of adversity within the family system.

## 6. Community-Level Effects on Intergenerational Transmission of ACEs

Biological and family-centered perspectives to understanding ACEs can be enriched by a social ecological viewpoint that integrates community-level influences. Recent efforts to adapt the original ACEs categories recognize the importance of the broader social ecology in children’s experiences of adversity, and they incorporate socioeconomic (i.e., financial hardship), school (i.e., peer victimization), community (i.e., community violence), and culturally relevant (i.e., discrimination, historical trauma) experiences [43,45,46]. A focus on community environments as a source of ACEs is especially important given that negative community forces, like racial discrimination in childhood, place youth at risk for behavioral problems [47] and psychological distress [48]. Similarly, researchers have proposed that the consequences of war and conflict (e.g., displacement, home destruction, and death of family members) should be considered ACEs given their known deleterious implications for children’s well-being [49,50]. Socioeconomic deprivation, peer victimization, and community violence are also powerful predictors of service need and poor health [43,45,51].

These community-level factors do not operate in isolation. Rather, research has documented relationships between ACEs and characteristics of a family’s greater community, including neighborhood affluence, disadvantage, disorder, and collective trauma [52,53,54]. To understand the relationships between community and family environments embedded within a historical framework, socioecological models posit that community violence and disadvantage permeate the family unit, augmenting stressors or decreasing supports to ultimately negatively impact children’s functioning [55,56]. Community violence and lack of neighborhood cohesiveness are associated with high continuity of ACEs from parents to children [42,57]. Similarly, maternal ratings of neighborhood disorder predict children’s direct exposure to ACEs, which, in turn, increases children’s problematic behavior, internalization of difficulties, and attention problems [58]. Similarly, caregiver exposure to political conflict and war predicts household IPV [59] and child maltreatment [60]. Further, community disadvantage may potentiate the detrimental effects of ACEs. For example, perinatal exposure to IPV was associated with infant disorganized attachment behaviors only among dyads living in highly disadvantaged neighborhoods [61]. Moreover, experiences of historical trauma, or collective traumatic experiences that occur over time, have been shown to impact psychological and physical health across generations [54]. Changes in gene expression via historical trauma that occur in tandem with maternal stress during pregnancy place children at an increased risk for subsequent ACEs [62].

Macro-level inequality may uniquely place families of color at risk for adverse outcomes. For example, mothers’ exposure to racism has been associated with higher risk for preterm delivery [63] and maternal depression [64], conferring risk for poor health outcomes in children. Structural racism contributes to significant health disparities in pregnancy-related mortality [65]. African American women in the United States are four times more likely than White women to die during childbirth [66], further amplifying adversity for a disproportionate number of Black children who grow up without their biological mothers.

Given that racism, poverty, and discrimination are demonstrated ACEs and barriers to health among families and children of color, we propose that intergenerational and community-level supports to prevent ACEs must look toward macro-level change. This recommendation aligns with a liberation psychology framework, which underscores the need for social resistance against oppressive forces to effectively address root causes of violence and foster individual and community healing [67,68].

## 7. Resilience in the Context of ACEs

The available literature emphasizes the many deleterious consequences of ACEs, with evidence that ACEs have rippling effects across individuals, families, and communities leading to poor health outcomes across generations. There is also research showing that many individuals, including children, fare well even in the midst of compounding adversity [69,70]. The ACEs literature has generally overlooked narratives of resilience, resulting in a lopsided and predominantly negative perspective on functioning following childhood adversity [71]. Yet, multifinality is apparent in the aftermath of ACEs, with observed diversity in outcomes and multiple trajectories of functioning based on the type and timing of childhood adversity [72]. For example, Schaefer and colleagues found that ACEs impacted individual resilience and posttraumatic growth among emerging adults, with younger age at first childhood adversity associated with lower levels of adaptive outcomes in adulthood [73]. Thus, as work in ACEs advances, it is essential to include a more robust examination of resilience, with specific focus on how resilience may manifest across generations rather than solely highlighting the negative ramifications of adversity. Such an intergenerational approach will promote strengths-based intervention for marginalized communities in which ACEs have historically been experienced at high levels.

There are divergent ways to conceptualize resilience, with some researchers viewing it as a relatively stable, innate characteristic or personal trait [74] and others defining it as an outcome or process of positive adaptation [75]. Contemporary perspectives model multisystemic resilience, which highlights dynamic connections across systems, including biological, psychological, social, and ecological [76]. Utilizing this framework with children experiencing adversity, Masten and Motti-Stefanidi found a multitude of interconnected factors within the individual child, families, schools, and communities that interact and work in concert to influence adaptive capacities among disaster-exposed youth [77]. A systems definition of resilience aligns with an intergenerational framework, as resilience can be transmitted across generations when it is conceptualized as existing both within and outside of the individual. The quality of these multiple reciprocating systems, and interactions among the systems, contributes to resilience following adversity [76].

The available ACEs research lends support to the multisystemic resilience model, demonstrating that processes within and across systems give rise to positive adaptation. Individual promotive factors include regulatory flexibility, problem-solving skills, and self-esteem [78,79]. For example, Schultz and colleagues examined adaptation over time and found that children exposed to maltreatment in early childhood exhibited more resilient functioning in early adolescence if they had higher problem-solving and daily living skills [79]. At the relational level, parenting competence, positive peer relationships, and caring adults (e.g., mentors, teachers) contribute to resilient outcomes for children [80,81]. More specifically, longitudinal data showed that children exposed to ACEs during the toddler and preschool years were more likely to follow a resilient trajectory and show competence in middle childhood if they had nurturing caregivers who provided warmth and cognitive stimulation [80]. Additionally, relational resources such as perceived parental care and caregiving competence have been uniquely associated with higher resilience in adults who experienced ACEs during their childhood [82]. Thus, from an intergenerational perspective, just as challenges can be passed down from one generation to the next, so too can strengths and adaptive functioning.

At the contextual level, positive school climate, a close-knit community, and safe neighborhoods have been linked to resilient outcomes for children exposed to ACEs [83,84]. More specifically, communities that allow children to safely play, explore, and maximize their capacities can cultivate adaptive outcomes and enhance positive functioning during childhood [81]. One study, for example, assessing the influence of ACEs in a population-based survey of US children found that youth with more school engagement and youth receiving family-centered medical care exhibited greater resilience [85]. The importance of community in supporting youth resilience is underscored by evidence that neighborhood involvement and cohesiveness may protect children from the detrimental effects of family dysfunction [86], as community resources and extrafamilial adults (e.g., mentors, coaches) may take on roles related to monitoring and supporting youth [87]. Community, therefore, may represent an important mechanism for disrupting the harmful effects of the cycle of intergenerational transmission of ACEs. The multisystemic resilience model highlights the necessity of accounting for interactions among these multiple systems when evaluating resilience [76].

This perspective shift from underscoring individual problems to highlighting ecological strengths is valuable to intervention science. Specifically, the multisystemic resilience model is a useful tool to understand how ACEs may affect access to resources and how these resources can be harnessed and enhanced via intervention. There is the potential to break the intergenerational transmission of adversity by developing resilience-promoting interventions.

## 8. Interventions to Mitigate the Effects of ACEs

Extant research demonstrating the relevance of intergenerational and trans-systemic processes in understanding resilience and maladaptation suggests that intervention frameworks should transition from focusing solely on the individual to broader conceptualizations of family, community, and cultural processes that represent root causes of the promulgation of ACEs and their negative effects. Moreover, programs need to not only address ACEs directly, but also address key biological, psychological, and social factors within the parent–child dyad that amplify risk. To this end, several promising practices have emerged that may provide important avenues for more comprehensive systems of care.

Programs delivered during pregnancy may be especially critical to address the potential of intergenerational ACEs transmission during the earliest stages of life [88]. Some existing programs, such as the *Integrated Behavioral Intervention*, have shown evidence of effectiveness in lowering behavioral risk factors, women’s victimization, and very-preterm birth [89,90]. The *Nurse Family Partnership Program* [91], rooted in social ecological and attachment theories, has also demonstrated effectiveness on a number of dimensions—most critically, in reducing risk for child maltreatment across various indicators. A recent review of interventions for violence-exposed pregnant women, however, noted that programs for pregnant women have often had difficulty establishing evidence of compelling and sustained effects across multiple dimensions of assessment, including both mental health and violence prevention [92].

Responding to this critical need for a comprehensive and developmentally informed intervention, the *Pregnant Moms’ Empowerment Program* (PMEP) was established [93]. The PMEP, which was adapted from the *Moms’ Empowerment Program* [94] and developed through iterative collaboration with pregnant women, service providers, and community stakeholders, foregrounds empowerment and resilience to underscore positive adaptation as the result of multisystemic processes. Specifically, adaptive functioning is enhanced by increasing mothers’ knowledge of and access to available resources across the social ecology, including individual, relational, and community-level supports. Consistent with an intergenerational framing, the PMEP facilitates group discussions on patterns of violence across the family system, the adverse effects of violence on mothers and their children, and available local resources related to safety, prenatal care, and early parenting. After promising pilot data, which demonstrated positive treatment effects on women’s depression and re-victimization, as well as infants’ socioemotional and language development [93], the efficacy of the PMEP is currently being evaluated in the context of a multisite randomized controlled trial in South Bend, IN and Memphis, TN. This research will assess the PMEP’s effectiveness in addressing maternal mental health, resilience, re-victimization, parenting sensitivity, and consequent infant developmental outcomes.

Intergenerational transmission of risk may also be addressed at other developmental junctures, and research has supported the importance of early, developmentally sensitive interventions. One study on responsive parenting interventions, for example, highlighted that parenting supports attuned to children’s developmental needs lead to different, and developmentally relevant, skill acquisition for participating parents [95]. For children who are experiencing psychopathology, many parenting programs for internalizing and externalizing behavior problems have achieved a strong evidence basis, with some research even suggesting their potential for positive epigenetic effects [96]. Importantly, parenting research and interventions in psychology have focused overwhelmingly on the role of biological mothers, but it is critical to recognize that children are likely to have other significant relationships with fathers, second parents, and other caregivers [97,98]. To more completely address the legacy and effects of adversity within families, interventions need to focus more broadly on familial and caregiver inclusivity; those that have done so have been met with promising effects [99,100].

It is also important to note that ACEs experienced by caregivers during their own childhood are associated with long-term, negative effects, including depression, posttraumatic stress, anxiety, and physical health problems during adulthood [4,5,6]. Further, caregiver ACEs may indirectly impact children’s adjustment via their consequent experiences of psychopathology [16,17]. As such, interventions aimed at promoting parental health and wellbeing are likely to have important trickle-down effects for children.

In addition, interventions should work to leverage known multisystemic protective and promotive factors for caregivers and children. Such work is necessarily multisectoral, engaging not only in the direct care and support of caregivers and children, but in assisting their connection to community resources, such as schools with strong support and social cohesion [83,84]. Further, through integrating programs and services in community settings, in concert with other supports for families, psychologists can additionally contribute to the healthful development of safe community spaces for families and children that may promote adaptive outcomes and enhance positive functioning [81].

Further, addressing systemic factors that can promote or inhibit the accessibility and effectiveness of care is an important target for intervention. One promising direction, for example, is the *Academic Centers for Excellence in Youth Violence Prevention*, which aims to facilitate community participation and mobilization to support the dissemination of evidence-based programs [101]. Other work has suggested the importance of intervention development and implementation practices more explicitly considering the impact of structural racism. These programs show the importance of multisectoral responses, leveraging context-specific intersecting risks, and confronting the effects of structural racism by educating implementers and separating from problematic institutions [102]. A coordinated and holistic response to intervention is likely critical in addressing structural inequity, as factors such as enhancing neighborhood quality, increasing income and employment opportunities, and improving education have all been shown to have positive health effects [103]. Finally, culturally sensitive treatment paradigms aimed at reducing the effects of historical trauma may promote healing and augment stressors that have contributed to the intergenerational transmission of ACEs [104].

## 9. Controversies and Future Directions

Over the past 20 years, research on children, trauma, and psychopathology has been inextricably shaped by Felitti and colleagues’ groundbreaking ACEs study [3]. Yet, the construct and measurement of ACEs employed in the original study and much of the subsequent research have not been without controversy [1]. Criticism abounds that the ACEs questionnaire should have been created with a more rigorous methodology (e.g., systematic review of the adversity literature) and that its psychometric properties require more thorough and sophisticated exploration [105]. In addition, there is much ambiguity about how comprehensive or constrained the definition of ACEs and scope of their sequelae should be. For example, as reviewed above, community factors hold the potential not only to expose children to ACEs, but also to exacerbate the intergenerational transmission of ACEs. Yet, the ACEs questionnaire omitted risk factors related to community violence, racial discrimination, social inequity, and historical trauma [54,106]. As a related problem, although the original ACEs study did not sufficiently measure all kinds of childhood adversity, the field has not yet come to a consensus about which adversities constitute an ACE.

This conceptual confusion has important ramifications for future ACEs research. An emerging body of literature shows differential effects from the maltreatment and household dysfunction domains of the ACEs questionnaire on mental health outcomes. For example, elevated socioemotional problems among young children were predicted by exposure to child maltreatment ACEs, but not household dysfunction ACEs [107]. Among adolescents, the only household dysfunction ACE that predicted adolescents’ depression, anxiety, and posttraumatic stress was witnessing partner violence against one’s mother, whereas all of the maltreatment ACEs significantly predicted negative mental health outcomes [108]. In samples of pregnant adult women, multiple studies have found that exposure to maltreatment ACEs predicted elevated posttraumatic stress symptoms (PTSS), whereas the household dysfunction ACEs did not, and in both studies, earlier exposure to child maltreatment predicted greater PTSS [109,110]. Similarly, McDonnell and Valentino found that, although both household dysfunction and child maltreatment ACEs predicted women’s depressive symptoms during the prenatal period, child maltreatment was a more highly significant predictor, and household dysfunction ACEs no longer significantly predicted depression after mothers gave birth [21]. These findings show that both the type of ACEs one experiences (i.e., child maltreatment more than household dysfunction) and the timing of exposure (i.e., in early childhood), as well as measurement of the outcome variable (i.e., perinatal vs. postpartum depression), matter in quantifying the relationship between ACEs and mental health outcomes. Overall, this emerging literature suggests that different ACEs are not equal in their predictive power of specific outcomes, and more nuanced investigations into the relationships between ACEs and both adverse and resilient outcomes are needed.

As we move into a new era of ACEs research, a variety of domains emerge as pressing areas for exploration. Identifying how cultural factors influence the impact of ACEs and the specific indicators that best reflect adversity during childhood is a critical next step. In research based in the United States, for example, unique patterns of ACEs exposure have been reported among Latino immigrant and Native American families, two groups that disproportionately experience socioeconomic disadvantage, historical trauma, and marginalization [46,111]. Findings suggest that additional ACEs, such as forced separation from family and involvement with immigration law enforcement, may be needed to represent the adverse experiences of children in these populations [111,112].

Regarding the intergenerational transmission of adversity, research on maternal ACEs abounds, but scant literature has included paternal or second parent’s ACEs as predictors of subsequent child ACEs and psychosocial functioning. Even less of the literature has examined the intergenerational transmission of ACEs via other caregivers (e.g., grandparents, aunts, uncles). Further, nearly all of the research in this area focuses on heterosexual couples; it is crucial for future research to explore intergenerational transmission of ACEs in same-sex families. There is also a lack of knowledge regarding how children come to reside in nonparental care (e.g., foster care) [113]. Future studies should explore how living in an alternative care setting may function as an ACE or how intergenerational transmission of ACEs from biological parents may be exacerbated by residence outside of the home. Further, very few studies have investigated the divergent trajectories of ACEs exposure on different children in the household. Evaluating the factors that explain differing developmental trajectories among siblings can inform our understanding of the disparate effects of ACEs on well-being across the lifespan, ranging from disease and psychopathology to resilience [114]. To date, however, researchers have not determined how intergenerational transmission of adversity is linked to differing developmental and psychological outcomes, both adaptive and maladaptive, in children in the same family. This may be a key next step for identifying how adversity is reflected in subsequent generations and what trajectories should be targeted for intervention.

Research also indicates that childhood experiences of ACEs are associated with future violence perpetration [115,116]. For example, individuals who report histories of abuse, neglect, and IPV exposure in childhood are more likely to perpetrate child abuse, engage in criminally violent behavior, and experience and perpetrate IPV in adulthood [115,117]. Additional work is warranted regarding what individual, relational, and community factors aid in breaking the “cycle of violence” in family systems with a history of ACEs. Finally, more research is needed to elucidate how the long-lasting effects of intergenerational adversity operate through causal pathways, with specific attention on mutable factors that may be amenable to intervention.

## 10. Conclusions

This review looked back on the past 20 years of ACEs research, with an eye toward the intergenerational impact of ACEs and multisystemic resilience following adversity. It is clear from this body of work that the burden of ACEs is much broader than individual suffering and impairment, and that disparities in health and well-being are partly rooted in the adversities experienced by previous generations. By taking a multilevel and intergenerational approach to conceptualizing ACEs, this review identified specific areas to target via intervention. We described how interventions, including those aimed at addressing intergenerational effects, may represent important evolutions in translational work on ACEs by focusing on the multiple, historic, and interconnected systems that perpetuate adversity across time. As the field of psychology emerges as a hub science, we can unite various biological, psychological, and sociological perspectives to inform our understanding of ACEs and our approach to strengths-based intervention.

## Data Availability

Not applicable.
